# Bacterial Isolates and Drug Susceptibility Pattern of Sterile Body Fluids from Tertiary Hospital, Northern Ethiopia: A Four-Year Retrospective Study

**DOI:** 10.1155/2019/5456067

**Published:** 2019-05-21

**Authors:** Ephrem Tsegay, Aregawi Hailesilassie, Haftamu Hailekiros, Selam Niguse, Muthupandian Saravanan, Mahmud Abdulkader

**Affiliations:** ^1^Department of Medical Microbiology and Immunology, School of Medicine, College of Health Science, Mekelle University, Mekelle, P.O. Box 1871, Ethiopia; ^2^Ayder Comprehensive Specialized Hospital laboratory, Mekelle University, Mekelle, P.O. Box 1871, Ethiopia

## Abstract

This study was conducted in Ayder comprehensive specialized Hospital, Mekelle, Northern Ethiopia, to determine the bacterial profiles and drug susceptibility pattern from body fluids. A total of 218 patients were investigated, of which 146 (67%) were males. The age of the study subjects ranged from 2 days to 80 years with 96(44%) in the age group of 15 years and above. The overall bacterial infection was 44 (20.2 %) of which gram positive bacteria were prevalent, 23 (52.3%) than gram negative bacteria 21 (47.7%). The predominantly isolated bacteria were* S. pneumonia, *followed by* K.pneumoniae, S. aureus*, and* E coli. *Multidrug resistance was observed in 12 (100%) of the isolated gram positive bacteria and in 6 (75%) of the isolated gram negative bacteria.

## 1. Introduction

Sterile body sites are those in which no bacteria or microbes exist as commensals when in a healthy state. This can be either pathological agents or contaminants from the skin in intensive care units [1]. Body fluids like ascitic, pleural, synovial fluids, cerebrospinal, and hydrocele are frequently received samples in the microbiology laboratory for culture in suspected infections [2]. These infections are associated with considerable morbidity and mortality [3]. Positive cultures are expected to be low because of less number of pathogens as well as prior administration of empirical antibiotics especially in intensive care units [4]. Recently World Health Organization and European Commission have recognized the importance of studying the emergence and the determinants of antibiotic resistance and the need for strategies for its control [5]. Reports from Centre for Disease Control Report suggested that strains are developing resistance to many antibiotics; risk practices are exposing patients for multidrug resistance putting peoples at increased risk for severe morbidity and there is a need for strong surveillance drug resistance [6].

Therefore, prevention of the emergence and dissemination of resistant organisms and their efficient management are critical for control of hospital infections. In addition, surveillance of antimicrobial susceptibility is necessary to combat the emergence of resistance [7, 8]. Due to the practice of empirical treatment in resource-constrained settings, the prevalence could be very high. Creating awareness of local antimicrobial susceptibility pattern and identifying the common bacterial pathogen is essential. Moreover for better management of patients and framing the antibiotic policy, understanding the knowledge of likely prevalent strains along with their antimicrobial resistance is essential [9–11].

In Ethiopia, few studies were conducted to review the bacterial profile from body fluids. However, these studies were targeting single bacteria from body fluids. Studies focusing on reviewing the profile of different bacteria from different body fluids are required [12, 13]. Hence, this study was aimed at identifying the bacterial isolates and determining the antimicrobial susceptibility patterns of the isolated bacteria from body fluids of patients attended Ayder Comprehensive Specialized Hospital.

## 2. Materials and Methods 

### 2.1. Study Design, Area, and Period

A retrospective study was conducted on the records of consecutive diagnoses on bacterial infection of sterile body fluids in the period from November 2013 to May 2016 at Ayder comprehensive specialized Hospital (ACSH), Mekelle, Northern Ethiopia. This study was evaluated and approved by the Research Ethics Review Committee (RERC) of College of Health Sciences, Mekelle University. Moreover, the letter of cooperation and permission were obtained from ACSH. The permission involves the permission to disseminate the findings of the study through the scientific workshop and publish in reputable journals.

### 2.2. Sampling and Study Population

A data abstraction format was prepared and both sociodemographic (like age and sex) and clinical data (a type of body fluid, isolated bacteria, antibiotic discs tested, and antibiotic sensitivity test result) were collected from the microbiology laboratory registration book. Data were abstracted from records of both inpatients and outpatients. Culture and sensitivity tests were done for all sterile body fluids (ascetic, synovial, cerebrospinal fluid, and pleural fluids). The positive cultures and their antibiotic susceptibility testing were performed under the guidelines of the Clinical and Laboratory Standard Institute (CLSI). The susceptibility to the following antimicrobial agents (OXOID, UK) was assessed: ampicillin, penicillin G, tetracycline, ceftriaxone, cotrimoxazole, amoxicillin-clavulanic acid, erythromycin, ciprofloxacin, chloramphenicol, gentamycin, norfloxacin, doxycycline, and nitrofrontine.

### 2.3. Quality Control

Reference strains of* S. aureus *(ATCC 25923),* E. coli *(ATCC 25922), and* P. aeruginosa *(ATCC 27853) were used as quality control for culture and susceptibility testing.

### 2.4. Data Analysis

Data were checked for completeness and accuracy and analyzed using SPSS version 21. The presence of association of independent variables bacterial isolation in the body fluids was determined using Chi-square. A p value of less than 0.05 was considered significant in all the analysis.

## 3. Results

### 3.1. Demographic Characteristics of the Study Subjects

During the study period, a total of 218 patients were investigated. Majority of the study participants, 146 (67%), were males, whereas 72 (33%) were females. The age of the study subjects ranged from 2 days to 80 years with majority 96 (44%) in the age group of 15 years and above.

### 3.2. Prevalence of Bacterial Infection

Out of 218 cultured sterile body fluid specimens, the overall bacterial isolates were 44 (20.2 %). From the patients investigated for bacterial infection 21 (47.7%) were from CSF, 12 (27.3%) from pleural, and 11 (25%) from other sterile body fluids (synovial fluid and ascetic fluid) being infected. Of the total 44 isolates, gram positive bacteria were prevalent, 23 (52.3%) than gram negative bacteria, 21 (47.7%). The predominantly isolated bacteria were* S. pneumonia with *11(25%) followed by* K.pneumoniae *5(11.4%),* S. aureus *4(9.1%),* E. coli *4(9.1%), and* P.aeruginosa *4(9.1%) (Table 1). We found a statistically significant association of specimen type with the prevalence (F-Test=9.073 and p-value=0.01). However, there was no significant association of bacterial isolation with sex (F-Test=0.277 and p-value=0.595) and age (F-test=1.702 and p-value=0.656).

### 3.3. Antimicrobial Susceptibility Testing

Among the gram positive bacteria (n=23), 66.7%, 66.7%, and 75% of the isolates were sensitive to amoxicillin-clavulanic acid, trimethoprim/sulphamethoxazole, and ceftriaxone, respectively. The resistance pattern of these isolates ranges from 16.7% for ceftriaxone to 78.6% for ampicillin.* S. pneumoniae,* which were the predominant isolates among gram positive bacteria 11 (47.8%), show susceptibility pattern of 4 (100%), 3 (100%), and 6 (75%) to amoxicillin-clavulanic acid, ciprofloxacin, and trimethoprim/sulfamethoxazole, respectively (Table 2). In gram negative bacterial isolates (n=21) showed a resistance rate of 86.8% to ampicillin. Resistance against gentamycin, norfloxacin, trimethoprim/sulfamethoxazole, chloramphenicol, nitrofurantoin, and amoxicillin-clavulanic acid was observed in the range of 45-75%. However, all gram negative bacterial isolates showed low-level resistance against ciprofloxacin (Table 3). Multidrug resistance was observed in 12 (100%) of the isolated gram positive bacteria and in 6 (75%) of the isolated gram negative bacteria.

## 4. Discussion

Sterile body fluids infection is one of the highly prevalent diseases in developing countries including Ethiopia. Specifically, the highest burden of meningococcal meningitis occurs in sub-Saharan Africa. [14, 15]. In this study that comprising 218 (CSF, pleural, ascitic and synovial body fluid) samples received in the microbiology laboratory, the percentage of positive cultures was 20.2% which has higher finding than studies done in India, 14.79% [16] and 14.78% [17]. The reason for this wide disparity in positivity rates of sterile fluids was attributed to differences in techniques, antibiotic use, or the prevalence of effusions caused by infective processes. Some of the variations are likely explained by the differences in the study population [14]. In our present study, 52.3% of infections were caused by gram positive bacteria and 47.7% by gram negative bacteria. The similar predominance of gram positive bacteria has been observed in previous studies conducted in the USA and India [17, 18]. The present study has shown* S.pneumoniae* as a predominant bacterial isolate which is in line with previous studies done in Northern parts of Yemen [19]. In contrast to the above observation, some studies in India [17] reported* S.aureus* to be the most common cause of sterile body infections.

The reported spectrum of microorganisms responsible for body fluid infection is varied and is modified by introduction of antibiotics, patient-specific factors such as surgical procedures, trauma, or underlying conditions, or by methodological factors, namely, the proper specimen collection, transport, and culture. For these reasons, several studies have found discordant results in the spectrum of pathogens causing these infections [20] Resistance to antimicrobial agents has been noted since the first use and is an increasing worldwide problem [21]. The present study revealed that gram negative bacterial isolates had shown a higher prevalence rate of resistance to the commonly prescribed antibiotics.* K. pneumoniae, E. coli, P. aeruginosa, Citrobacter *spp* and Acinetobacter *spp. isolates were resistant to Ampicillin (100%) and this implies that ampicillin cannot be used as empirical therapy for sterile body infections particularly in the study area. Resistance against gentamycin, norfloxacin, trimethoprim/sulfamethoxazole, chloramphenicol, nitrofurantoin and amoxicillin-clavulanic acid was observed in the range of 45-66.7%. On the other hand, very low levels of resistance were observed against ciprofloxacin (26.3%). This observation corroborates with other studies reporting antimicrobial susceptibility patterns of Gram negative bacteria isolated from sterile body fluids [17, 18]

Among the Gram positives bacteria, 66.7%, 66.7%, and 75% of the isolates were sensitive to amoxicillin-clavulanic acid, trimethoprim/sulphamethoxazole, and ceftriaxone, respectively. The resistance pattern of these isolates ranges from 16.7% for ceftriaxone to 78.6% for ampicillin.* S. pneumoniae,* which were the predominant isolates among Gram-positive bacteria 11 (47.8%), show susceptibility pattern of 4 (100%), 3 (100%), 6 (75%) to amoxicillin-clavulanic acid, ciprofloxacin, and trimethoprim/sulphamethoxazole, respectively. This finding is comparable to previous studies [17, 18]. However, this study shows the effectiveness of amoxicillin-clavulanic acid, ciprofloxacin, and ceftriaxone against Gram positive bacteria is reduced when compared with the previous comparable study [17, 18]. This may be due to the frequent prescription of amoxicillin-clavulanic acid for empiric therapy. Since it is a retrospective study, the study population was not systematically selected, and since a relatively low number of cultures were performed over the study time-period the results may not be truly representative. In addition, only aerobic cultures were performed thus limiting identification to anaerobic pathogens. Nevertheless, the data are of value with respect to antimicrobial susceptibility of sterile body fluid pathogens in Ethiopia.

## 5. Conclusion

In conclusion, the yield of body fluids cultures is usually low. CSF and pleural fluid showed the high bacterial prevalence and predominantly isolated bacteria were* S. pneumonia, *followed by* K. pneumoniae, E. coli, P. aeruginosa, Citrobacter *spp., and* Acinetobacter *spp. Low culture positivity may be due to the presence of anaerobic or fastidious organisms with lack of enrichment techniques and prior antibiotic administration. Regular monitoring of the prevalent pathogenic organisms and their sensitivities will aid the clinician's appropriate selection of antibiotic therapy to prevent the development of antimicrobial resistance.

## Figures and Tables

**Table 1:** Distribution of bacterial etiologic agents from body fluids.

Isolated Bacteria	CSF	Pleural fluid	OBF¶	Total (n=218)
	N (%)	N (%)	N (%)	N (%)
*Gram positive*	*11(48)*	*6(26)*	*6(26)*	*23(52.3)*
*S. pneumoniae*	7	4	0	11
S.*aureus*	1	1	2	4
CoNS*∗*	2	1	0	3
*Enterococcus Spp*	1	0	1	2
*Streptococcus spp*	0	0	1	1
*Micrococcus lutes*	0	0	1	1
*Kocuriarosea*	0	0	1	1
*Gram negative*	*10(47.6)*	*6(28.6)*	*5(23.8)*	*21(47.7)*
*K.pneumoniae*	2	3	0	5
*E. coli*	4	0	0	4
*P.aeruginosa*	1	1	2	4
*Klebsielaspp*	1	0	2	3
*Citrobacterspp*	0	1	1	2
*Proteus spp*	0	1	0	1
*Acitinobacterspp*	1	0	0	1
*H.influenza*	1	0	0	1
Total	*21(47.7)*	*12(27.3)*	*11(25)*	*44(100.0)*

CoNS*∗*:- Coagulase negative Staphylococcus, ¶OBF=synovial and ascetic fluid

**Table 2:** Antimicrobial susceptibility pattern of gram-positive bacteria (n=23) isolated from body fluids.

Bacterial Isolates	Pattern	CIP	CRO	AMC	C	P	TE	SXT	E	AMP
		N (%)	N (%)	N (%)	N (%)	N (%)	N (%)	N (%)	N (%)	N (%)
*S.pneumoniae* (n=11)	R	0(0.0)	0(00.0)	0(0.0)	3(50.0)	1(10.0)	1(10.0)	1(12.5)	4(57.1)	5(62.5)
	I	0(0.0)	1(30.0)	0(0.0)	3(50.0)	3(30.0)	2(20.0)	1(12.5)	0(0.0)	0(0.0)
	S	3(100)	6(60.0)	4(100)	0(0.0)	6(60.0)	7(70.0)	6(75.0)	3(42.9)	3(37.5)

S.*aureus*(n=4)	R	0(0.0)	1(50.0)	1(100)	0(0.0)	2(66.7)	0(0.0)	1(33.3)	1(25.0)	0(0.0)
	I	0(0.0)	0(0.0)	0(0.0)	0(0.0)	0(0.0)	1(25.0)	0(0.0)	1(25.0)	0(0.0)
	S	1(100)	1(50.0)	0(0.0)	1(100)	1(33.3)	3(75.0)	2(66.7)	2(50.0)	0(0.0)

CoNS(n=3)	R	0(0.0)	0(0.0)	0(0.0)	0(0.0)	1(100)	0(0.0)	1(50.0)	1(50.0)	0(0.0)
	I	0(0.0)	0(0.0)	0(0.0)	0(0.0)	0(0.0)	0(0.0)	0(0.0)	0(0.0)	0(0.0)
	S	0(0.0)	0(0.0)	0(0.0)	1(100)	0(0.0)	3(100)	1(50.0)	1(50.0)	0(0.0)

*Enterococcus Spp*(n=2)	R	2(100)	1(100)	1(100)	0(0.0)	1(100)	2(100)	0(0.0)	2(100)	2(100)
	I	0(0.0)	0(0.0)	0(0.0)	0(0.0)	0(0.0)	0(0.0)	0(0.0)	0(0.0)	0(0.0)
	S	0(0.0)	0(0.0)	0(0.0)	1(100)	0(0.0)	0(0.0)	0(0.0)	0(0.0)	0(0.0)

*Streptococcus spp*(n=1)	R	0(0.0)	0(0.0)	0(0.0)	0(0.0)	1(100)	1(100)	0(0.0)	1(100)	1(100)
	I	0(0.0)	0(0.0)	0(0.0)	0(0.0)	0(0.0)	0(0.0)	0(0.0)	0(0.0)	0(0.0)
	S	0(0.0)	1(100)	0(0.0)	0(0.0)	0(0.0)	0(0.0)	1(100)	0(0.0)	0(0.0)

*Micrococcus lutes*(n=1)	R	0(0.0)	0(0.0)	0(0.0)	0(0.0)	0(0.0)	0(0.0)	0(0.0)	1(100)	0(0.0)
	I	0(0.0)	0(0.0)	0(0.0)	0(0.0)	0(0.0)	0(0.0)	0(0.0)	0(0.0)	0(0.0)
	S	0(0.0)	1(100)	0(0.0)	1(100)	0(0.0)	0(0.0)	0(0.0)	0(0.0)	0(0.0)

*Kocuriarose* (n=1)	R	1(100)	0(0.0)	0(0.0)	0(0.0)	0(0.0)	1(100)	1(100)	0(0.0)	0(0.0)
	I	0(0.0)	0(0.0)	0(0.0)	0(0.0)	0(0.0)	0(0.0)	0(0.0)	0(0.0)	0(0.0)
	S	0(0.0)	0(0.0)	0(0.0)	0(0.0)	0(0.0)	0(0.0)	0(0.0)	0(0.0)	0(0.0)

Total (n=23)	R	3(42.9)	2(16.7)	2(33.3)	3(33.3)	6(37.5)	5(23.8)	4(26.7)	10(57.8)	11(78.6)
	I	0(0.0)	1(8.3)	0(0.0)	3(33.3)	3(18.8)	3(14.3)	1(6.6)	1(5.8)	0(0.0)
	S	4(57.1)	9(75.0)	4(66.7)	3(33.3)	7(43.7)	13(61.9)	10(66.7)	6(35.3)	3(21.4)

*CIP* = ciprofloxacin *CRO* = ceftriaxone *AMC* = amoxicillin-clavulinic acid, *C* = Chloramphenicol, *P* = penicillin *TE* = tetracycline, *SXT*= trimethoprim/sulfamethoxazole. *E* = erythromycin *AMP* = ampicillin.

R = Resistant I = Intermediate S = Sensitive

**Table 3:** Antimicrobial susceptibility pattern of gram-negative bacteria (n=21) isolated from body fluids.

Bacteria Isolates	Pattern	CIP	NOR	CN	AMC	C	F	AMP	SXT	DOX
		N (%)	N (%)	N (%)	N (%)	N (%)	N (%)	N (%)	N (%)	N (%)
*K.pneumonae* (n=5)	R	1(0.0)	2(50.0)	2(40.0)	1(50.0)	2(66.7)	1(33.3)	3(100)	2(66.7)	4(80.0)
	I	1(33.3)	0(0.0)	0(0.0)	0(0.0)	1(33.3)	1(33.3)	0(0.0)	0(0.0)	0(0.0)
	S	3(66.7)	2(50.0)	3(60.0)	1(50.0)	0(0.0)	1(33.3)	0(0.0)	1(33.3)	1(20.0)

*E. coli* (n=4)	R	2(100)	2(50.0)	1(33.3)	2(66.7)	1(33.3)	1(50.0)	2(100)	0(0.0)	2(50.0)
	I	0(0.0)	0(0.0)	1(33.3)	0(0.0)	0(0.0)	0(0.0)	0(0.0)	0(0.0)	0(0.0)
	S	2(0.0)	2(50.0)	1(33.3)	1(33.3)	2(66.7)	1(50.0)	0(0.0)	0(0.0)	2(50.0)

*P. aeruginosa* (n=4)	R	1(25.0)	2(100)	2(50.0)	0(0.0)	1(50.0)	0(0.0)	2(100)	1(50.0)	3(100)
	I	1(25.0)	0(0.0)	2(50.0)	0(0.0)	0(0.0)	0(0.0)	0(0.0)	1(50.0)	0(0.0)
	S	2(50.0)	0(0.0)	0(0.0)	0(0.0)	1(50.0)	0(0.0)	0(0.0)	0(0.0)	0(0.0)

*Klebsielaspp* (n=3)	R	1(33.3)	0(0.0)	2(66.7)	2(66.7)	1(100)	1(100)	3(100)	1(50.0)	1(0.0)
	I	0(0.0)	0(0.0)	0(0.0)	0(0.0)	0(0.0)	0(0.0)	0(0.0)	0(0.0)	0(0.0)
	S	2(66.7)	1(100)	1(33.3)	1(33.3)	0(0.0)	0(0.0)	0(0.0)	1(50.0)	0(0.0)

*Citrobacterspp* *(n=2)*	R	2(100)	0(0.0)	1(50.0)	2(100)	0(0.0)	0(0.0)	2(100)	0(0.0)	0(0.0)
	I	0(0.0)	0(0.0)	0(0.0)	0(0.0)	0(0.0)	0(0.0)	0(0.0)	0(0.0)	0(0.0)
	S	0(0.0)	0(0.0)	1(50.0)	0(0.0)	0(0.0)	0(0.0)	0(0.0)	1(100)	0(0.0)

*Proteus spp* *(n=1)*	R	0(0.0)	0(0.0)	0(0.0)	0(0.0)	1(100)	0(0.0)	0(0.0)	0(0.0)	0(0.0)
	I	0(0.0)	0(0.0)	1(100)	0(0.0)	0(0.0)	0(0.0)	1(100)	0(0.0)	0(0.0)
	S	1(100)	1(100)	0(0.0)	1(100)	0(0.0)	1(100)	0(0.0)	0(0.0)	0(0.0)

*Acinetobacter spp.* *(n=1)*	R	0(0.0)	1(100)	1(100)	1(100.0)	0(0.0)	1(100)	1(100)	0(0.0)	0(0.0)
	I	0(0.0)	0(0.0)	0(0.0)	0(0.0)	0(0.0)	0(0.0)	0(0.0)	0(0.0)	0(0.0)
	S	1(100)	0(0.0)	0(0.0)	0(0.0)	0(0.0)	0(0.0)	0(0.0)	0(0.0)	0(0.0)

*H. influenzae* *(n=1)*	R	0(0.0)	0(0.0)	0(0.0)	0(0.0)	0(0.0)	0(0.0)	0(0.0)	0(0.0)	1(100)
	I	0(0.0)	0(0.0)	0(0.0)	0(0.0)	0(0.0)	0(0.0)	0(0.0)	0(0.0)	0(0.0)
	S	1(100)	1(100)	1(100)	0(0.0)	1(100)	1(100)	1(100.0)	0(0.0)	0(0.0)

Total (n=21)	R	5(26.3)	7(50)	9(45.0)	8(66.7)	6(54.6)	5(55.6)	13(86.8)	4(50.0)	11(78.6)
	I	2(10.5)	0(0.0)	4(20.0)	0(0.0)	1(9.0)	0(0.0)	1(6.6)	1(12.5)	0(0.0)
	S	12( 63.2)	7(50.0)	7(35.0)	4(33.3)	4(36.4)	4(44.4)	1(6.6)	3(37.5)	3(21.4)

*CIP* = ciprofloxacin *NOR* = norfloxacin*CN* = gentamicin *AMC* = amoxicillin-clavulinic acid. *CRO* = ceftriaxone *C* = chloramphenicol. *F* = Nitrofurantoin *AMP* = ampicillin *SXT* =trimethoprim/sulfamethoxazole. *R* = Resistant *I* = Intermediate *S* = Sensitiv

## Data Availability

The data used to support the findings of this study are available from the first author and corresponding author upon request.

## Abbreviations

CLSI: Clinical and Laboratory Standard Institute
CoNS: Coagulase negative staphylococcus
CSF: Cerebrospinal fluid
SPSS: Statistical Package for Social Sciences
WHO: World Health Organization.
